# Diagnostic accuracy of dopaminergic imaging in prodromal dementia with Lewy bodies

**DOI:** 10.1017/S0033291718000995

**Published:** 2018-04-25

**Authors:** Alan J. Thomas, Paul Donaghy, Gemma Roberts, Sean J. Colloby, Nicky A. Barnett, George Petrides, Jim Lloyd, Kirsty Olsen, John-Paul Taylor, Ian McKeith, John T. O'Brien

**Affiliations:** 1 Institute of Neuroscience, Newcastle University, Campus for Ageing and Vitality, Newcastle upon Tyne NE4 5PL, UK; 2 Nuclear Medicine Department, Leazes Wing, Royal Victoria Infirmary, Richardson Road, Newcastle upon Tyne NE1 4LP, UK; 3 Department of Psychiatry, University of Cambridge School of Clinical Medicine, Box 189, Level E4 Cambridge Biomedical Campus, Cambridge CB2 0SP, UK

**Keywords:** Alzheimer's, DLB, dopaminergic imaging, FP-CIT, MCI

## Abstract

**Background:**

Dopaminergic imaging has high diagnostic accuracy for dementia with Lewy bodies (DLB) at the dementia stage. We report the first investigation of dopaminergic imaging at the prodromal stage.

**Methods:**

We recruited 75 patients over 60 with mild cognitive impairment (MCI), 33 with probable MCI with Lewy body disease (MCI-LB), 15 with possible MCI-LB and 27 with MCI with Alzheimer's disease. All underwent detailed clinical, neurological and neuropsychological assessments and FP-CIT [^123^I-N-fluoropropyl-2*β*-carbomethoxy-3*β*-(4-iodophenyl)] dopaminergic imaging. FP-CIT scans were blindly rated by a consensus panel and classified as normal or abnormal.

**Results:**

The sensitivity of visually rated FP-CIT imaging to detect combined possible or probable MCI-LB was 54.2% [95% confidence interval (CI) 39.2–68.6], with a specificity of 89.0% (95% CI 70.8–97.6) and a likelihood ratio for MCI-LB of 4.9, indicating that FP-CIT may be a clinically important test in MCI where any characteristic symptoms of Lewy body (LB) disease are present. The sensitivity in probable MCI-LB was 61.0% (95% CI 42.5–77.4) and in possible MCI-LB was 40.0% (95% CI 16.4–67.7).

**Conclusions:**

Dopaminergic imaging had high specificity at the pre-dementia stage and gave a clinically important increase in diagnostic confidence and so should be considered in all patients with MCI who have any of the diagnostic symptoms of DLB. As expected, the sensitivity was lower in MCI-LB than in established DLB, although over 50% still had an abnormal scan. Accurate diagnosis of LB disease is important to enable early optimal treatment for LB symptoms.

## Background

Prodromal dementia may include different patterns of symptom onset and presentation (McKeith *et al*. 2016), but here we use it synonymously with mild cognitive impairment (MCI), i.e. for the cognitive presentation. Biomarkers are likely to be important for improving diagnostic accuracy in MCI and are included in the published criteria for MCI due to Alzheimer's disease (AD) (Albert *et al*. 2011; Dubois *et al*. 2014). In dementia with Lewy bodies (DLB), clinical studies report the diagnostic value of dopaminergic imaging, especially FP-CIT [^123^I-N-fluoropropyl-2*β*-carbomethoxy-3*β*-(4-iodophenyl)] nortropane single-photon emission computed tomography (O'Brien *et al*. 2014), and we reported similar accuracy of FP-CIT validated against autopsy (Thomas *et al*. 2017).

Only a few studies, all retrospective (e.g. Auning *et al*. 2011; Chiba *et al*. 2012), have assessed the clinical features of MCI stage of Lewy body (LB) disease [summarised in a review by (Donaghy *et al*. 2015)]. In our previous report on this cohort, we found that using supportive neuropsychiatric symptoms, as defined in the 2017 DLB diagnostic criteria (McKeith *et al*. 2017), usefully distinguished MCI with Lewy body disease (MCI-LB) from MCI with AD (MCI-AD) (Donaghy *et al*. 2018). In contrast, although MCI-LB subjects scored lower on tests of attention, visuospatial function and verbal fluency, these did not accurately differentiate MCI-LB from MCI-AD (Donaghy *et al*. 2018). Others have examined the neurocognitive profile and reported that amnestic MCI predicts conversion to AD whilst non-amnestic MCI is associated with DLB (Ferman *et al*. 2013). However, there have been no reports of the utility of dopaminergic imaging in MCI.

We therefore investigated the diagnostic value of FP-CIT dopaminergic imaging in a prospective study of a cohort of MCI followed up for over a year. We hypothesised that FP-CIT would have similar specificity but lower sensitivity at the MCI stage where the impact on nigrostriatal neurones will be less than at dementia stage, and that this would result in an overall improvement in diagnostic confidence of MCI-LB as estimated using likelihood ratios.

## Methods

### Individual symptoms and final diagnosis by consensus panel

Details of recruitment, clinical assessment and clinical diagnoses have been reported previously (King *et al*. 2017; Donaghy *et al*. 2018). Briefly, study subjects were over 60 years of age and recruited from memory clinics, specialist dementia services, elderly medicine clinics and neurology clinics in the North East of England. They had been diagnosed with MCI in these services and were eligible for the study if at some stage they were reported to have at least one clinical symptom suggesting the possible presence of DLB, e.g. autonomic symptoms, visual disturbances, olfactory impairment and mood changes, as well as any indication of the presence of core and suggestive features of DLB.

All subjects provided written informed consent in accordance with our ethical approval, following which they had a detailed standardised neuropsychological assessment by a research nurse or psychologist and a thorough diagnostic medical assessment by a board certified old age psychiatrist (PD), including blood sampling, lumbar puncture and neurological examination. After this, subjects underwent FP-CIT imaging (see below for details). This report focuses on FP-CIT findings in this MCI population. We have previously reported the clinical symptoms and neuropsychological findings in our earlier paper (Donaghy *et al*. 2018). So we report only key summary descriptive neuropsychological and clinical data here. Study subjects were reviewed after a year and the detailed clinical assessment was repeated.

An expert consensus clinical panel (AT, PD, J-PT) reviewed all the clinical assessment data to confirm that subjects met National Institute on Aging-Alzheimer's Association (NIA-AA) criteria for MCI without considering aetiology (Albert *et al*. 2011). The consensus panel also rated the presence or absence of each of the four core clinical LB symptoms characteristic of DLB in the fourth consensus report of the DLB consortium [cognitive fluctuations, complex visual hallucinations, clinical parkinsonism, clinical probable rapid eye movement (REM) sleep behaviour disorder (RBD)] (McKeith *et al*. 2017). This consensus panel approach has been validated previously against autopsy diagnoses (McKeith *et al*. 2000, 2007).

As described previously (King *et al*. 2017; Donaghy *et al*. 2018), subjects were allocated in one of the three clinical diagnoses: probable MCI-LB (NIA-AA MCI plus two or more of the four clinical core LB symptoms), possible MCI-LB (MCI plus one of the four symptoms) and MCI-AD (MCI with none of these four symptoms and evidence of decline which was a characteristic of AD with no evidence for another aetiology, i.e. they met the additional NIA-AA criterion of ‘aetiology of MCI consistent with AD pathophysiologic process’). The ‘1-year rule’ was applied so that no subjects had had evidence of Parkinsonism for more than a year before the onset of their cognitive decline. Assignment to these diagnostic categories was based on the information from both baseline and annual follow-up clinical evaluations. The consensus panel assessment of both MCI and LB symptoms, and allocation to diagnostic category at both time points, was done blind to FP-CIT results.

### Acquisition of FP-CIT images

Three to six hours following a bolus intravenous injection of 185 MBq of ^123^I-FP-CIT (DaTSCAN, GE Healthcare, UK), patients were scanned (25 min) using a double-headed *γ* camera (Siemens Symbia S) fitted with a low-energy high-resolution parallel hole collimator. One hundred and twenty (60 per detector) 25 s views over a 360° orbit were acquired on a 128 × 128 matrix with a zoom of 1.23× giving a pixel size of 3.9 mm × 3.9 mm. Image processing and display were then performed on a Hermes workstation (Hermes Medical Solutions, Stockholm, Sweden). Images were reconstructed without attenuation correction using filtered back projection and a Butterworth filter (order 10, cut-off 1.3 cycles/cm). Transverse sections were then manually reoriented to correct for any head tilt and to provide a consistent display.

### Visual rating of FP-CIT images

Visual assessment of all scans was undertaken blind to clinical diagnosis and information by four raters: one consultant medical physicist experienced in nuclear medicine reporting, one experienced neuroimaging analyst and certified old age psychiatrists (JL, SJC, AT, PD). Prior training for this task involved all raters reading details of the visual rating scale in advance and having previous experience in the use of the scale (Benamer *et al*. 2000), as well as visual inspection of an independent dataset of 10 scans ranging from normal to markedly abnormal to allow raters to see the full range of scan appearances. Our group has extensive experience in FP-CIT rating and, e.g. had inter-rater reliability (intra-class correlation coefficient 0.93) when reporting FP-CIT in a recent study in people with dementia (Lloyd *et al*. 2018). Then, for the rating session, all scans were independently randomised and blinded from clinical data before being presented to each rater. Using an identical colour map scaled to the maximum voxel count, transverse sections were displayed with the cross-platform image viewer ‘MRIcron’ (https://www.nitrc.org/projects/mricron). Scans were rated independently by each panel member using an established four-category FP-CIT visual rating procedure (Benamer *et al*. 2000), which has also showed diagnostic value in the differential diagnosis of DLB and AD (O'Brien *et al*. 2004). Briefly: grade 0 (bilateral tracer uptake in caudate and putamen and largely symmetric); grade 1 (asymmetric uptake with normal or almost normal putamen activity in one hemisphere with more marked reduction in the contralateral putamen); grade 2 (significant bilateral reduction in putamen uptake with activity confined to the caudate) and grade 3 (significant bilateral reduction in uptake affecting both the caudate and the putamen). After rating all scans, any scan where there was no complete agreement between all raters was then subsequently reviewed by all four raters together where a consensus rating was agreed. All scans were rated as normal or abnormal and where abnormal were graded as 1–3. In addition to primary visual reading, all scans underwent semi-quantitative analysis using DaTQUANT software (v1.0, GE Healthcare), comparing study scans against age-adjusted norms, to ensure no FP-CIT scans with significant balanced striatal loss (i.e. abnormal scans) were inadvertently rated as normal. Following this semi-quantitative analysis, no scans were reclassified as abnormal.

### Statistical analysis

The Statistical Package for Social Sciences software (SPSS version 23) was used for statistical evaluation. For group comparisons, χ^2^ tests were used for categorical variables, and for continuous variables, we tested for normality of distribution and *t* tests or Mann–Whitney tests were used. Diagnostic accuracy of FP-CIT (sensitivity, specificity and overall accuracy) was calculated from standard 2 × 2 frequency tables, and 95% confidence was calculated using Minitab (version 16.1). Likelihood ratios were then calculated to estimate the potential-added diagnostic value of using FP-CIT imaging.

## Results

**Fig. 1.**
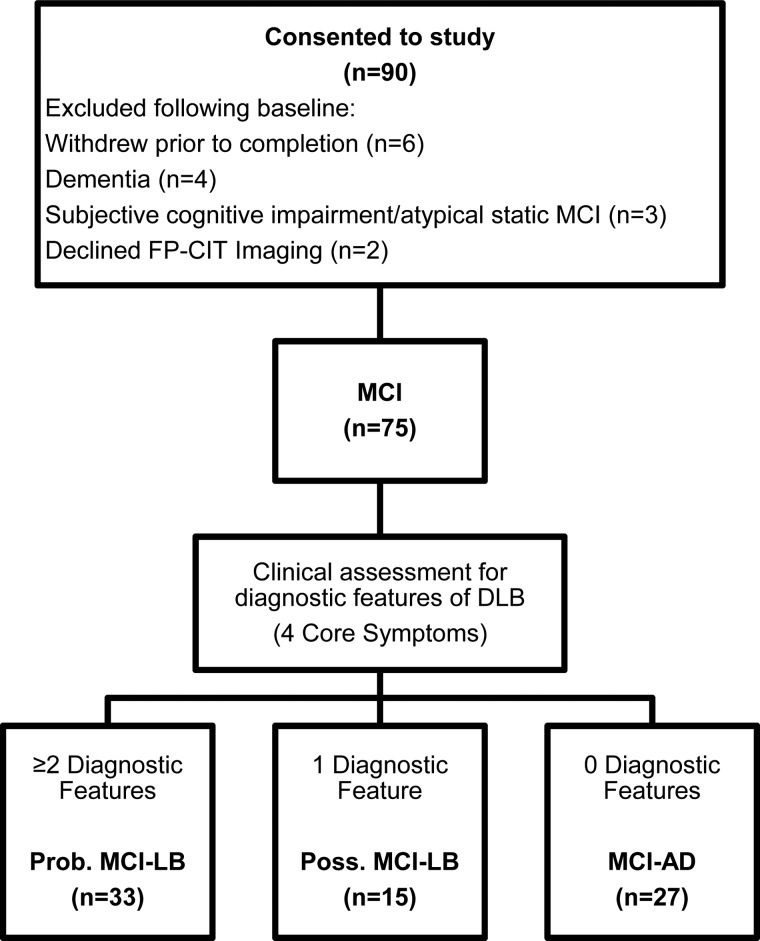
Classification of subjects. Subjects with two or more diagnostic features were classified as MCI-LB. Subjects with no diagnostic features were classified as MCI-AD. Subjects with one diagnostic feature were classified as possible MCI-LB. Prob./Poss MCI-LB, probable/possible MCI with Lewy bodies; MCIAD, MCI due to Alzheimer's disease.

A total of 90 subjects consented to the study. However, 15 did not meet entry criteria after full baseline assessment or withdrew before or declined the FP-CIT scan. Thus, 75 subjects met all criteria and completed baseline assessments and FP-CIT imaging (see Fig. 1).

After assessment by the diagnostic panel, 33 subjects were found to have two or more core LB symptoms and were classified as probable MCI-LB. Fifteen had one symptom and were classed as possible MCI-LB. Twenty-seven were found not to have any diagnostic LB symptoms and were classed as MCI-AD. In these cases, any symptoms indicating core features of LB disease, which had been identified by the referring service, were not confirmed by the detailed research assessment, e.g. a reported tremor was not parkinsonian and there were no other features of Parkinson's disease (PD); or the symptoms indicating possible LB disease, e.g. constipation or anosmia, were present but no core or suggestive diagnostic features of LB disease were present. No subject had evidence of neuroleptic sensitivity.

Table 1 shows the demographic and clinical characteristics of the three groups. There were no differences across the groups in age, gender or severity of cognitive impairment, with the mean Mini-Mental State Examination (MMSE) score being over 26 and mean Clinical Dementia Rating (CDR) score being <0.5, consistent with the MCI status of study subjects. The overall physical health status of the subjects was similar on the Cumulative Illness Rating Scale for Geriatrics (CIRS-G), with the great majority of subjects scoring 0–2 on this scale (meaning they had only mild disability or moderate disability controlled by first-line therapy), and similar to previous reports at the MCI stage (e.g. Borson *et al*. 2010).

**Table 1.** Demographic and clinical data for the three MCI groups

	Probable MCI-LB^a^ *N* = 33	Possible MCI-LB^a^ *N* = 15	MCI-AD^a^ *N* = 27	^a^Probable MCI-LB = A; possible MCI-LB = B; MCI-AD = C
Age (years)	75.0 (7.5)	75.9 (8.3)	77.2 (7.8)	*F*_(2,72)_ = 0.60, *p* = 0.55
Gender (M:F)	22:11	9:6	11:16	*X* = 4.17, df = 2, *p* = 0.12
MMSE	26.5 (1.8)	25.9 (2.9)	26.5 (2.2)	*F*_(2,72)_ = 0.41, *p* = 0.67
ACE-R	79.3 (7.7)	78.0 (13.4)	79.7 (11.2)	*F*_(2,72)_ = 0.14, *p* = 0.87
CDR	0.45 (0.15)	0.43 (0.18)	0.44 (0.16)	*F*_(2,72)_ = 0.1, *p* = 0.91
CIRS-G	8.9 (4.2)	12.2 (5.1)	8.8 (3.8)	*F*_(2,72)_ = 3.7, *p* = 0.03 A *v*. B = 0.02, A *v*. C = 0.93, B *v*. C = 0.02
IADL	14.6 (5.1)	12.3 (3.4)	9.9 (2.1)	*F*_(2,55)_ = 7.7, *p* = 0.001, A *v*. B = 0.12, A *v.* C = *p* < 0.001, B *v*. C = *p* = 0.05
UPDRS	26.7 (15.8)	15.5 (11.8)	14.6 (6.9)	*F*_(2,72)_ = 8.3, *p* = 0.001, A *v*. B, *p* = 0.01, A *v*. C, *p* < 0.001, B *v*. C, *p* = 0.74
H&Y (stages: 0/1/2/3/4/5)	18/0/7/7/1/0	13/0/1/1/0/0	27/0/0/0/0/0	*X* = 18.5, df = 6, *p* = 0.005, A *v*. B = *p* = 0.19, A *v*. C = *p* = 0.001, B *v*. C = *p* = 0.15
ESS	10.8 (5.4)	7.8 (3.7)	4.4 (3.9)	*F*_(2,72)_ = 14.3, *p* < 0.001, A *v*. B = *p* = 0.03, A *v*. C = *p* < 0.001, B *v*. C = *p* = 0.01
NPI	13.4 (9.5)	14.3 (12.6)	6.1 (6.8)	*F*_(2,64)_ = 4.8, *p* = 0.012, A *v*. B = *p* = 0.80, A *v*. C = *p* = 0.003, B *v*. C = *p* = 0.04
NPI distress	7.8 (7.2)	7.9 (6.9)	3.0 (4.5)	*F*_(2,64)_ = 5.0, *p* = 0.009 A *v*. B = 0.64, A *v.* C = 0.008, B *v*. C = 0.003
GDS	4.4 (3.5)	3.6 (3.3)	2.2 (2.2)	*F*_(2,72)_ = 4.2, *p* = 0.019 A *v*. B = 0.45, A *v*. C = 0.005, B *v*. C = 0.09
Medication at baseline number (percentage)				
Anti-dementia	17 (51.5)	0 (0)	7 (25.9)	*X* = 13.3, df = 2, *p* = 0.001
Anti-parkinsonian	7 (21.2)	1 (6.6)	0 (0)	*X* = 7.3, df = 2, *p* = 0.03
Antipsychotic	2 (6.1)	0 (0)	0 (0)	*X* = 2.61, df = 2, *p* = 0.27
Antidepressant	11 (33.3)	7 (46.7)	11 (40.7)	*X* = 0.85, df = 2, *p* = 0.65

MMSE, Mini-mental State Examination; ACE-R, Addenbrooke's Cognitive Examination – Revised; CDR, Clinical Dementia Rating scale; CIRS-G, Cumulative Illness Rating Scale for Geriatrics; IADL, Instrumental Activities of Daily Living scale; UPDRS, Unified Parkinson's Disease Rating Scale – Part III; H&Y, Hoehn and Yahr rating scale; ESS, Epworth Sleepiness Scale; NPI, Neuropsychiatric Inventory; GDS, Geriatric Depression Scale.

a Probable MCI-LB = A; possible MCI-LB = B; MCI-AD = C.

As expected, the MCI-LB groups had higher scores on the Hoehn and Yahr scale and Movement Disorder Society Unified Parkinson's Disease Rating Scale (MDS-UPDRS) and had more impairment on the Instrumental Activities of Daily Living (IADL) scale due to their combined physical and mental impairments. Consistent with the diagnosis, the MCI-LB groups had higher sleepiness (Epworth Sleepiness Scale), depression (Geriatric Depression Scale) and behavioural disturbance (Neuropsychiatric Inventory).

### FP-CIT visual rating

Table 2 shows that abnormal FP-CIT findings were found in 61% of probable MCI-LB and 40% of possible MCI-LB. Three MCI-AD subjects were rated as having an abnormal FP-CIT scan (all were classified as grade 1). Abnormal scans were more frequent in the MCI-LB groups than in the MCI-AD group whether assessed as normal *v.* abnormal or by grade on the visual rating scale. But the probable and possible MCI-LB were not different from each other in the frequency of abnormal FP-CIT. Of the 26 subjects with abnormal FP-CIT scans in the MCI-LB groups, only 12 (46%) had clinical parkinsonism on neurological examination, and thus only 41% of people overall with abnormal scans had parkinsonism (12 of 29 abnormal scans). The mean *κ* for inter-rater reliability was 0.59, and for intra-rater reliability it was 0.73.

**Table 2.** FP-CIT ratings for probable MCI-LB, possible MCI-LB and MCI-AD

	Probable MCI-LB^a^ *N* = 33	Possible MCI-LB^a^ *N* = 15	MCI-AD^a^ *N* = 27	Probable MCI-LB = A; possible MCI-LB = B; MCI-AD = C
Consensus panel rating of FP-CIT number abnormal (percentage)	20 (61.0)	6 (40.0)	3 (11.1)	*X* = 15.4, df = 2, *p* < 0.001 A *v*. B *X* = 1.76, df = 1, *p* = 0.18 A *v*. C *X* = 15.4, df = 1, *p* < 0.001 B *v*. C *X* = 4.78, df = 1, *p* = 0.03
Consensus panel semi-quantitative grades of FP-CIT rating: 0/1/2/3 (percentage)	13/7/9/4 (39/21/27/12)	9/5/1/0 (60/33/7/0)	24/3/0/0 (89/11/0/0)	*X* = 22.4, df = 6, *p* = 0.001 A *v*. B *X* = 5.48, df = 3, *p* = 0.14 A *v*. C *X* = 17.4, df = 3, *p* = 0.001 B *v*. C *X* = 5.32, df = 3, *p* = 0.07

a Probable MCI-LB = A; possible MCI-LB = B; MCI-AD = C

Overall the sensitivity of visually rated FP-CIT imaging to detect combined possible and or probable MCI-LB was 54.2% [95% confidence interval (CI) 39.2–68.6], with a specificity of 89.0% (95% CI 70.8–97.6), giving an overall diagnostic accuracy of 66.6% (95% CI 54.8–77.0). The sensitivity in probable MCI-LB was 61.0% (95% CI 42.5–77.4), and in possible MCI-DLB it was 40.0% (95% CI 16.4–67.7).

The likelihood ratio for MCI-LB was therefore 4.9, indicating that FP-CIT dopaminergic imaging may be a clinically important test.

After unblinding, we reviewed the FP-CIT images and DaTQUANT semi-quantification data on the three abnormal scans in patients diagnosed with MCI-AD and confirmed that these were indeed abnormal scans. We were also able to review the progression of these subjects over the 1-year follow-up, and only one developed any core or suggestive features of LB disease by the 1-year review. This subject developed cognitive fluctuations. Two of these subjects consented to having cardiac metaiodobenzylguanidine (MIBG) scans in another study (the third, who had developed cognitive fluctuations, declined) and both of these scans were abnormal.

By the time all surviving subjects had had at least 1 year of clinical review of diagnosis, the mean duration of follow-up was 1.5 years, with 27 subjects having had a second year review and nine a third year review. Twenty-one subjects had progressed to dementia, all to the expected disease dementia subtype [14 with probable DLB (McKeith *et al*. 2017), three with possible DLB (McKeith *et al*. 2017) and four with probable AD dementia (McKhann *et al*. 2011)], and five had died. Of those who had died, four had consented to brain tissue donation. Detailed neuropathological examination of these four subjects found neuropathology consistent with their clinical diagnoses. Two had been diagnosed as MCI-LB and both had neocortical LB disease and met the McKeith criteria for DLB. The other two had MCI-AD and both met the NIA-AA criteria for neuropathological change due to AD.

## Discussion

We report here for the first time, the performance of dopaminergic imaging at the MCI stage of cognitive decline. Compared with MCI-AD, dopaminergic imaging had an 89% specificity for MCI-LB and a positive likelihood ratio of 4.9, i.e. a positive (abnormal) scan increases the absolute probability of having MCI-LB by about 30% (McGee, 2002), which is a clinically important increase. This suggests that the use of FP-CIT imaging at the MCI stage in people with any symptom characteristic of LB disease may be clinically useful. As hypothesised, because of less advanced nigrostriatal degeneration in earlier disease, we also found that at this stage of illness the sensitivity was lower (54.2%) compared with 80% at the dementia stage (O'Brien *et al*. 2014).

It is important to note that a large proportion (40%) of patients with only a single-core diagnostic symptom of DLB (cognitive fluctuations, motor parkinsonism, complex visual hallucinations and REM sleep behaviour disorder) had abnormal FP-CIT at this early stage, with the proportion with abnormal scans rising as expected when more such symptoms were present. It is also important that at this MCI stage, most subjects with abnormal scans did not have clinical evidence of parkinsonism (only 41%), demonstrating that biomarkers such as FP-CIT are able to detect disease in advance of clinical detection of parkinsonism and have an important role to play in early diagnosis of dementia. This figure of 41% compares with 83% of people with abnormal scans having clinical parkinsonism at the dementia stage (O'Brien *et al*. 2004), consistent with the earlier stage of assessment in this study.

These findings therefore support the use of FP-CIT imaging in people with MCI who have any of these core features of DLB in order to improve the detection of LB disease and distinguish it from AD. The value of a diagnostic test depends on its setting. For the identification of potential disease in the community settings, a high sensitivity is important for screening procedures. But in specialist settings, it is the specificity that is more important and a specificity of >80% has been suggested (Postuma *et al*. 2015). The 89% specificity here supports the value of FP-CIT imaging in distinguishing MCI-LB from MCI-AD.

Previous work has reported that FP-CIT imaging improves diagnostic accuracy compared with clinical diagnosis alone (Walker *et al*. 2007) and, whilst most studies in dementia have compared the more certain diagnosis of probable DLB with AD, two reports have indicated the value of FP-CIT in the less certain diagnosis of possible DLB, with diagnosis validated by clinical follow-up as in this study (O'Brien *et al*. 2009; Walker *et al*. 2014). Our findings suggest that FP-CIT has a utility at the MCI stage as well, and the future progression and diagnoses of our possible LB-MCI group are of particular interest. Early and accurate diagnosis of LB disease is important for the same reasons as early diagnosis of AD and also because it can prevent the fluctuations that are intrinsic to the disease being mistaken for delirium, strongly cautious against using antipsychotics and facilitates early identification and treatment of motor symptoms, dysautonomia and other characteristic non-psychiatric symptoms (NICE, 2006).

It is possible that the sensitivity, specificity, diagnostic accuracy and likelihood ratios may be even better. Of the three MCI-AD subjects with abnormal scans, one has now developed cognitive fluctuations and the other two have since had abnormal cardiac MIBG suggesting that their scans may not be false positives. It may be that both of these biomarkers are picking up, in these patients, LB disease before any LB clinical symptoms are manifested. The high specificity of FP-CIT in this study is similar to that reported at the dementia stage, but it is important to remember that FP-CIT imaging is not a specific marker of synuclein pathology and that other diseases, e.g. frontotemporal lobar degeneration, can cause abnormal scans (Tiraboschi *et al*. 2016; Thomas *et al*. 2017). Thus, we cannot exclude the fact that some of our patients may have early frontotemporal dementia or other rarer neurodegenerative diseases. Furthermore, it is our standard practice not to recruit people with evidence on structural imaging of infarcts in the basal ganglia as these may also cause false-positive FP-CIT scans, and we recommend that the clinicians should conduct structural scans before FP-CIT imaging where such cerebrovascular disease is suspected.

In addition, at this early stage, it is arguably inappropriate to say that the sensitivity of FP-CIT is low because many people with LB disease elsewhere, e.g. in neocortical and limbic areas, are likely to have insufficient LB disease involvement in the substantia nigra to produce an abnormal scan. Even at the dementia stage, we found 10% of people with confirmed LB disease had no significant nigral involvement (Thomas *et al*. 2017). Thus, whilst an abnormal FP-CIT scan strongly supports the presence of LB disease even at this early stage of illness, a normal scan is probably more likely to be a false negative than at the dementia stage and therefore does not rule out LB disease.

This study benefited from being a prospective analysis, from all subjects having a detailed clinical assessment by an experienced physician using validated rating scales, from consensus panel diagnosis and consensus panel rating of all diagnostic symptoms for DLB and by the FP-CIT ratings being conducted by an experienced panel of raters and confirmed by semi-quantification software, which has been demonstrated to improve the accuracy of ratings (Nicastro *et al*. 2017). The intra-rater and inter-rater *κ* values were lower than in some reported studies (e.g. Seibyl *et al*. 2014). This reflects the greater difficulty in rating subjects whose scans are at an earlier disease stage and where more, therefore, have borderline abnormal scans. Furthermore, unlike other FP-CIT studies, we had no subjects who are generally easier to rate, such as those with confirmed PD and healthy controls. The experienced FP-CIT raters when rating the study scans were aware of how much more difficult such scans were compared with those in people with more advanced diseases (clinical PD and DLB) and healthy controls. For example, we had inter-rater reliability (intra-class correlation coefficient 0.93) when reporting FP-CIT in a recent study in people with dementia (Lloyd *et al*. 2018). A key limitation of this study is that the subjects were largely identified from specialist memory and dementia services and so are not representative of people either in primary care or in other specialised secondary care settings, e.g. movement disorder clinics. In such settings, the specificity of FP-CIT is lower (Tiraboschi *et al*. 2016). All study subjects also had some feature or features at recruitment initially suggestive of LB disease and so MCI-AD patients, whilst fulfilling NIA-AA criteria after robust assessment, may not be representative of the wider MCI-AD population. However, it is likely that in such a sample of MCI-AD, the specificity of FP-CIT will be even higher due to a lower risk of contamination with ‘silent’ LB disease cases, and thus the performance of FP-CIT will be correspondingly greater.

The conversion rate to dementia has been at the expected rate of 10–15% per annum for the AD and possible MCI-LB groups but much higher (about 40%) in the probable MCI-LB in this cohort. This supports the study population as consisting of people with neurodegenerative disease, and suggests that the more rapid decline of people with DLB compared with AD (Mueller *et al*. 2017) applies also to this earlier MCI stage. The fact that people converted to the expected dementia subtype provides some validation of the accuracy of the MCI subtype diagnoses, and is in harmony with our use of diagnostic criteria consistent with those for DLB in the recent fourth report of the DLB consortium. Furthermore, all four cases who have come to autopsy had neuropathology diagnoses that agreed with their MCI disease diagnosis, providing some further reassurance about the diagnostic accuracy in this study. But until more diagnoses are validated against autopsy, they are necessarily less certain than those made at the dementia stage where the diagnostic criteria have had such tissue validation.

The fourth revision of the international consensus DLB criteria (McKeith *et al*. 2017) were used as the basis for the MCI-LB diagnoses in this report. This paper deals with the revised criteria for the dementia stage of cognitive decline associated with LB disease. We were able to apply these new consensus DLB criteria core features to our MCI subjects and so the MCI-LB criteria we used are consistent with these revised DLB criteria because they elevate clinical RBD (study subjects did not have polysomnography (PSG) to confirm RBD) to sit alongside the previous three core diagnostic features as symptoms for MCI-LB diagnosis. The report also acknowledges the need for studies such as this which will provide data for evidence-based criteria for improving the diagnosis of LB disease at the MCI stage of the illness in the future.
